# The complete mitochondrial genome of *Formosania galericula* (Cypriniformes: Gastromyzontidae)

**DOI:** 10.1080/23802359.2021.1997125

**Published:** 2021-11-12

**Authors:** Tian-jiang Chu, Jia-jun Zhou, Ying-ying Wang, Kai Liu

**Affiliations:** a Institute of Fishery Science, Hangzhou Academy of Agricultural Sciences, Hangzhou, China; b Zhejiang Forest Resource Monitoring Center, Hangzhou, China; c Wetland Museum of China, Hangzhou, China

**Keywords:** *Formosania galericula*, mitochondrial genome, next-generation sequencing, phylogeny

## Abstract

This study determined the complete mitochondrial genome of *Formosania galericula* (Cypriniformes: Gastromyzontidae) from Zhejiang, China, for the first time. The complete mitochondrial genome of *F. galericula* was sequenced to be 16,555 bp in length. The genome contained 13 protein-coding genes, 22 transfer RNAs, two ribosomal RNAs, two central non-coding regions (the control region and the origin of light strand replication), identical to most other vertebrates. Phylogenetic analysis highly supported that *F. galericula* was close to *Crossostoma lacustre* and the genus *Vanmanenia* fish. However, *F. galericula* was not firstly clustered with *Formosania chenyiyui* but after the genus *Vanmanenia* fish. These data would explain the evolutionary mechanisms and biogeography of the family Gastromyzontidae and is helpful for the conservation of genetics and stock evaluation for *F. galericula*.

*Formosania galericula* (Zhang and Wang 2011) is a species of the family Gastromyzontidae of the order Cypriniformes, found only in Zhejiang, China, according to FishBase (Froese and Pauly 2021). Zhang and Wang first discovered this species in 2011 in the Baishanzu National Nature Reserve of Zhejiang, China (Zhang and Wang 2011). The fish is very similar to *Formosania fasciolata* in morphology. The main difference between *F. galericula* and *F. fasciolata* is that the longest rostral barbel are equal in length to the diameter of the eye, with two bright spots behind the sides of the dorsal fin, absence of spots on the other fins, and naked area in the abdomen extends to ventral fin base (Zhang and Wang 2011). At present, there is no report on the mitochondrial genome of *F. galericula*. Here, we report the complete mitochondrial genome of *F. galericula* for the first time and evaluate the phylogenetic performance among Gastromyzontidae fish.

*F. galericula* was harvested from Zhejiang Wuyanling National Nature Reserve of China (119°38'8.56″E, 27°41'29.01″N), and deposited at the National Original Breeding Farm of black Amur bream from China's Qiantang River (120°07′21.99″E, 30°08′35.53″N). The total genomic DNA from the fin tissue (assigned as LBTQ202103) was extracted by the phenol-chloroform extraction method (Green and Sambrook 2012). After the genomic DNA was quantified, the DNA was sonicated using a Covaris M220. The sheared DNA fragments were purified and used to construct a sequencing library and subjected to next-generation sequencing (NGS). The NGS was performed by Origingene Bio-pharm Technology CO., Ltd (Shanghai, China). The mitochondrial genome of *F. galericula* was obtained by sequence assembly on the NGS data using NOVOPlasty ver. 4.3.1 (Dierckxsens et al. 2017). The accession was registered GenBank under accession numbers MZ662822. The annotation process was completed using the MITOFISH prediction server (Iwasaki et al. 2013).

The complete mitochondrial genome of *F. galericula* from Zhejiang Wuyanling National Nature Reserve is 16,555 bp in length and comprises 22 tRNAs, two ribosomal RNAs, 13 protein-coding genes (PCGs), and two central non-coding regions. The base composition of the mitochondrial genome of *F. galericula* is 29.31% A, 24.99% T, 28.61% C, 17.09% G, with an AT content of 54.30%. Most *F. galericula*'s genes and RNAs are encoded on the heavy strand (H-strand) except for *ND*6 and eight tRNAs encoded on the light strand (L-strand). Within the genome, all the 13 PCGs include the normal start codon ATG except for *CO*1, which is initiated with GTG. However, the stop codons of the 13 PCGs differ, these terminating with TAG, TAA, TA- or T–. The origin of light strand replication (OL), which extends up to 31 nucleotides, is identified in the WANCY region. The second non-coding region, the control region (D-loop), is located between the tRNA-Pro and tRNA-Phe with 892 bp in length. The phylogenetic tree of *F. galericula* is shown in Figure 1, drawn by IQ-TREE ver. 2.1.3 with the complete mitochondrial genomes, under the TIM2 + F + I + G4 substitution model (Minh et al. 2020). The reliability of the phylogenetic tree was tested by the Ultrafast Bootstrap method (repeated 1 000 times) (Hoang et al. 2018). SH-aLRT values and Ultrafast Bootstrap values were given in percentages. The phylogenetic analysis clearly showed that *F. galericula* (MZ662822) clustered firstly with *Crossostoma lacustre* (M91245, AP010774) into a branch, and they grouped with the genus *Vanmanenia* fish with high support except for *Vanmanenia lineata* (KY352774). *Crossostoma* and *Formosania* are different names for the same genus (Novák et al. 2006). Therefore, it is normal for *F. galericula* and *C. lacustre* to be in the same branch. However, it should be noted that *F. galericula* was not firstly clustered with *Formosania chenyiyui* (MK135435), but after the genus *Vanmanenia* fish, and finally clustered with *V. lineata*. In addition, *Pseudogastromyzon fasciatus* (KP866867) are in the same clade as the genus *Vanmanenia* fish, requiring attention.

**Figure 1.**
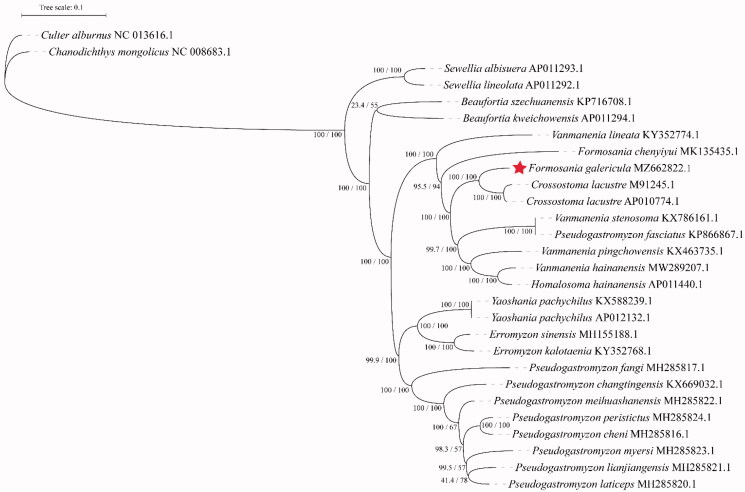
Phylogenetic tree of *Formosania galericula* inferred using the maximum likelihood method based on the mitochondrial genomes. Values are shown at each node of the tree, correspond to the SH-aLRT test values and Ultrafast Bootstrap value given in percentages.

## Data Availability

The data that support the findings of this study are openly available in National Center for Biotechnology Information (NCBI) at https://www.ncbi.nlm.nih.gov under accession no. MZ662822. The associated BioProject, SRA, and BioSample numbers are PRJNA750522, SRX11610055, and SAMN20462687, respectively.
